# 

**Published:** 1916-05

**Authors:** 


					﻿PUBLISHED MONTHLY.
M ’ J v.	• -f
SUBSCRIPTION $1.00
ATLANTA
JOURNAL-RECORD
OF MEDICINE
! Atlanta Medical and Surgical Journal, Established 1855.	) LQrrl Vp3
and Southern Medical Record, Established 1870.	vJiO I CO I
Vol. LXIII
Atlanta, Ga.,	1916
No.
				

